# Applications of Telemedicine in the Middle East and North Africa Region: Benefits Gained and Challenges Faced

**DOI:** 10.7759/cureus.26611

**Published:** 2022-07-06

**Authors:** Mohamed R Abouzid, Shorouk M Elshafei, Ibrahim Elkhawas, Mohamed K Elbana

**Affiliations:** 1 Internal Medicine, Baptist Hospitals of Southeast Texas, Beaumont, USA; 2 Internal Medicine, Mansoura University Faculty of Medicine, Mansoura, EGY; 3 Internal Medicine, Steward Carney, Dorchester, USA; 4 Internal Medicine, Aberdeen Royal Infirmary, Aberdeen, GBR

**Keywords:** covid-19, teletrauma, telepathology, teleophthalmology, teledermatology, telecardiology, teleradiology, telemedicine

## Abstract

Information and communication technology has left a print on all fields of life, including medicine and the health care system. Telemedicine is the perfect way to ensure adequate healthcare delivery to all people at any time, particularly during pandemics, regardless of any geographic or economic considerations. This article investigates the different types, categories, and benefits in addition to the barriers to telemedicine implementation, especially in the Middle East and North Africa (MENA) region. After a thorough review of medical literature related to telemedicine using PubMed, Google Scholar, and some other gray literature, it has been found that telemedicine has been involved in almost all medical specialties with a positive influence on healthcare delivery and medical education and research. It had a major role during the COVID-19 pandemic. However, many obstacles prevent its proper application and need to be addressed carefully by the government and relevant authorities. Due to the rapidly growing population, unequal distribution of healthcare services, and social distancing of the COVID-19 pandemic, the role of telemedicine has become increasingly essential. Regarding medical education and research, telemedicine facilitates the exchange of information and ideas between physicians and professionals from all over the world, bringing these various minds together on a single platform.

## Introduction and background

Telemedicine refers to physicians and other medical professionals delivering healthcare services through information and communication technologies that enable medical professionals and patients to exchange valid data and information related to the diagnosis, treatment, and prevention of several diseases and provide a good opportunity for patient counseling and health promotion. In addition to physician-patient interaction, telemedicine is a great way for physician-physician interaction in the form of continued medical education, teleconferences, and research.

## Review

Historical perspective of telemedicine

Telemedicine, over decades, has had different forms according to the available technological facilities. The earliest published article about telemedicine was in 1906 when ECG was transmitted over the telephone and the ECG inventor published a paper about telecardiograms [1]. In the early 1960s, the National Aeronautics and Space Administration (NASA) adopted the idea of telemedicine as an important element in their space flights. They started to provide their spacecraft and spacesuits with devices for telemedicine to monitor and keep in touch with astronauts in space. In 1964, the Nebraska Psychiatric Institute used television links to form a two-way connection with the physicians at Norfolk State Hospital, which was 112 miles away. In 1967, The Logan International Airport in Boston built in-house medical stations that are linked to Massachusetts General Hospital for providing consultation services to patients at the airport. These consultations were done through microwave audio and video links. In 1967 also, Kenneth Bird established one of the first telemedicine clinics at Massachusetts General Hospital, which provided occupational and emergency health services to employees and travelers at Boston's Logan International Airport, which was three miles away from the hospital. In 1972, the USA Department of Health, Education, and Welfare approved funding for seven telemedicine projects across different states that were renewed and another two projects were funded the following year. Over the past decades, telemedicine is evolving and experiencing major breakthroughs along with Internet and information technology development and increased availability and access to computers, laptops, and mobile phones [2-3].

Types of telemedicine

The three major types of telemedicine, based on the timing of the information sent, include [4]:

(1) Store-and-Forward: This type refers to storing the data such as medical images and biosignals after acquiring it from a patient and sending it to the receiver. The sender and receiver are not present or interacting together at the same time. The patient’s medical record is an essential component of this transfer. This type of telemedicine is commonly used in the medical fields of pathology, radiology, and dermatology.

(2) Remote Monitoring: Also known as self-monitoring or self-testing. This type refers to monitoring the health and clinical signs of a patient remotely using a wide variety of technological devices. This practice is commonly used in the management of chronic diseases such as cardiovascular disease, diabetes mellitus, and asthma. This type is cost-effective and enhances patient satisfaction; however, it carries some risk of getting inaccurate results of tests done by patients themselves.

(3) Real-Time Interactive Services: This type involves face-to-face communication between patient and healthcare provider with obtaining the medical history, consultation, discussing the management plan, and counseling.

According to the interaction between the individuals, telemedicine is classified into two categories [4]:

1. Health professional to health professional

2. Health professional to patient

The Middle East and North Africa (MENA) region faces several challenges that make telemedicine the best option to deliver the best patient health care. In 2018, the population of the MENA region was estimated at about 578 million and the most populous country in this region is Egypt representing 17% of all MENA population. Due to this fast-growing population and diverse demographics, the healthcare services are not evenly distributed among all areas of each country, and sometimes, it is difficult to deliver healthcare services to people who are living in rural or underprivileged areas. Healthcare facilities and well-equipped hospitals tend to be concentrated in big cities. In addition, transportation is another problem experienced by those living in rural or remote areas making it an obstacle for them to get higher healthcare levels in big cities.

Furthermore, we cannot ignore that the COVID-19 pandemic has shed the light on the importance and effectiveness of telemedicine as a reliable cost-effective tool to deliver patient education and consultation and also to continue medical education for health professionals. Due to the COVID-19 pandemic, telemedicine has become popular among the public more than any time in the past and people have started to realize its usefulness.

Categories of telemedicine and benefits

Teledermatology

Using telecommunication technology for the evaluation and management of patients with various skin problems has been demonstrated to be beneficial. It can produce a high degree of patient satisfaction in terms of transportation and waiting time spent in clinics. In addition, dermatologists are also satisfied with the effectiveness and diagnostic reliability of this practice either in the form of store and forward or real-time interaction [5-9]. However, skin cancer is still a debatable part of teledermatology. A study showed that the diagnostic accuracy of teledermatology in cases of skin cancer is inferior to that of direct clinical diagnosis with high sensitivity (98%) but low specificity (43%) [10-11].

Teleradiology

Teleradiology refers to transferring different radiological images (X-rays, CT, MRI, positron emission tomography (PET) scan, single-photon emission computerized tomography (SPECT)/CT, US) from one place to another [12]. It is the most commonly used category of telemedicine representing approximately 50% of telemedicine applications either in a synchronous (real-time interaction) or asynchronous (store and forward) mode [13]. Nowadays, unlike the way followed in the past, the image reviewer is able to get access to remote servers to view the radiological images. In the MENA region, there are several medical centers in isolated remote areas that lack experienced radiographers whose main role is the initial evaluation of the patient radiographic findings in addition to the limited access of a large population to big hospitals. This problem can lead to failure to reach the correct diagnosis and consequently deterioration of the patient's condition. Hence, teleradiology and synchronous teleconsultations play a significant role in these circumstances [14]. Despite the efficacy and cost-effectiveness of teleradiology, software, hardware, and video-conferences tools that are needed to transmit the radiological images are still highly costly and this represents the main obstacle against teleradiology application in many countries of the MENA region [15-16]. Apart from the unaffordable cost, the issues related to confidentiality of patient information, security, quality of transmitted images, sufficient training to use these complex devices, and periodic maintenance of the equipment should be addressed in the future for better and wider implementation of teleradiology in all the MENA region countries [17-19].

Telepathology

The MENA region suffers from an evident shortage of pathologists. In Africa, it was reported that there is one pathologist for every 500,000 people or more. In some countries, the ratio can exceed one pathologist for every five million people [20]. Telepathology can solve this issue through telecommunications between pathologists in different locations exchanging their experiences and sharing possible diagnoses in addition to promoting medical education and research. According to the American Telemedicine Association telepathology is defined as a form of communication between medical professionals that includes the transmission of pathology images and associated clinical information for the purpose of various clinical applications including, but not limited to, primary diagnoses, rapid cytology interpretation, intraoperative and second opinion consultations, ancillary study review, archiving, and quality activities [21].

There are four different forms of telepathology [22]:

1. Static telepathology: Refers to the transmission of pre-captured digital images (snapshots) through e-mail or storing them on a shared server then they can be reviewed by another consulting pathologist.

2. Whole slide imaging (WSI): Refers to digitization (scanning) of glass slides producing high-resolution digital slides so that the other pathologist can check the whole specimen at a range of magnifications.

3. Nonrobotic telepathology: Refers to the real-time transmission of live images via a video-calling platform, e.g., Zoom, Facetime, etc., to consulting pathologists without giving them control over the display.

4. Robotic telepathology: Similar to the nonrobotic form but the consulting pathologists have control over the display.

The critical barriers against the implementation of telepathology include the high cost of the required equipment, information technology-related problems in addition to the medico-legal issues related to liability of physicians, and confidentiality [23-24].

Teleophthalmology

Using telecommunications technology and digital medical devices to provide continuous eye care for those living in rural or underserved areas of the MENA region can have a substantial impact on improving the condition and quality of life and prevent the development of complications for those suffering from eye diseases.

Diabetes mellitus is one of the most common and serious healthcare issues in the MENA region. According to the International Diabetes Federation, the estimated prevalence of diabetes in 2019 was 55 million adults (ages 20-79), and it is growing rapidly. According to the American Academy of Ophthalmology, six out of the 10 countries with the highest diabetes prevalence worldwide are Middle Eastern countries (Saudi Arabia, United Arab Emirates, Kuwait, Bahrain, Lebanon, and Oman). Diabetic retinopathy is one of the most common and feared complications of diabetes that can end up in blindness. The prevalence of diabetic retinopathy is variable (19% in the United Arab Emirates, 20% in Egypt, and reaching 64% in Jordan).

In view of the previously mentioned statistical facts, teleophthalmology has a significant role in the ophthalmologic care of diabetic patients through frequent, low-cost, easily accessible screening for diabetic retinopathy and monitoring their cases remotely before the eye pathology deteriorates reaching the irreversible stage. This practice has been found to be reliable and effective and improves the compliance of diabetic patients to annual eye screening [25-28].

Teleophthalmology has also been found to be as reliable and effective in the measurement of visual acuity as that done by an optometrist in the office [29]. Moreover, teleophthalmology has proven its accuracy in diagnosing different causes of chronic blurred vision [30]. The application of teleophthalmology has a remarkable effect on the assessment and management of several eye diseases. It has enabled primary care physicians to properly manage over two-thirds of patients' vision complaints, increase the resolution rates of cases of refractive errors, and consequently decrease the number of referrals to specialists [31].

Telepsychiatry

It is also known as telemental health or E-mental health. Mental health is a challenging issue worldwide and in most of the MENA region in particular, since many individuals have insufficient awareness or education about it. Telemedicine can also be applied in the field of psychiatry and mental health in the form of providing psychiatric assessment, therapy (individual therapy, group therapy, and family therapy), patient education and medication management, and routine follow-up meetings, especially for those in remote hard-to-reach regions [32]. One of the examples is using video-conferencing technology to do cognitive-behavior therapy (CBT), supportive therapy, group therapy for depression and anxiety disorders, and trauma-focused therapies [33-34]. Moreover, it provides psychiatrists with opportunities for medical education and research. Most telepsychiatry application is in the form of synchronous (real-time interaction). Telepsychiatry is considered the most commonly applied category of telemedicine in developed countries [35]. Its application has shown effectiveness and reliability in psychiatric assessment, management, outcomes (clinical outcome, quality of life, and adherence), cost-effectiveness, patient compliance, and satisfaction of both patient and psychiatrist [36].

Teletrauma and Wound Care

Telemedicine has been reported to exert major benefits during pre-hospital and post-discharge management of trauma care, mainly in rural underserved areas. Pre-hospital care includes the transmission of a patient's video, medical images, ECG, and oxygen saturation. Post-discharge care includes the transmission of images and videos of the wound, ulcer, or burn to the physician for continuous monitoring and management of the condition accordingly. Such application to trauma and wound care results in preventing most trauma-related complications, improving the quality of life, and reducing mortality and morbidity [37-38].

Telecardiology

Telecardiology may include three domains: pre-hospital, in-hospital, and post-hospital applications [39]. Pre-hospital telecardiology has demonstrated efficacy in either the management of remote patients with acute coronary syndrome or in supporting primary care physicians with decision-making. Compared to a prospective control group, ECG-teleconsultation offers faster triage, shorter door-to-needle times, and shorter in-hospital delays in ST-elevation myocardial infarction (STEMI) patients [40-41]. When patients were brought directly to percutaneous coronary intervention (PCI) facilities, bypassing local hospitals, sending a pre-hospital 12-lead ECG straight to the attending cardiologist's mobile phone reduced the door-to-balloon time by more than one hour [42]. 

Most applications of in-hospital telecardiology refer to real-time echocardiogram transmissions between rural minor hospitals and tertiary care centers, specifically for the diagnosis or exclusion of congenital heart disease in neonates [39]. Moreover, numerous studies suggest that post-hospital telecardiology improves outcomes and decreases re-admissions or outpatient visits in patients with heart failure, arrhythmias, or implanted devices [43-44].

Telemedicine as a rescue solution in the COVID-19 era

During the COVID-19 pandemic, with its consequences of lockdown and social isolation, telemedicine is a key option and the perfect solution to continuously provide essential health care services as well as reduce the risk of infection spread. Individuals with chronic illnesses like diabetes, cardiovascular disease, respiratory disease, and psychiatric disorders, as well as pregnant women, can benefit most and receive a good level of healthcare through telemedicine, especially those who are at higher risk of morbidity and mortality from COVID-19 [45]. Mild and moderate COVID-19 cases can be managed and monitored via telemedicine. Some countries in the MENA region, such as Egypt, Saudi Arabia, and the United Arab Emirates, have implemented this strategy in managing and monitoring asymptomatic contacts and mild and moderate COVID-19 cases that do not require hospitalization. Such a strategy was quite effective in reducing the load on hospitals and physicians and decreasing the risk of infection spread by mild or moderate cases [45].

After discussing the benefits of each category of telemedicine, the obstacles that stop the implementation of telemedicine include [46-47]:

1. Lack of information and communication technology (ICT) infrastructure: Lack of ICT infrastructure, such as good internet connection and ICT technicians, in many regions especially rural areas, and the high cost is depriving many people of getting the benefit of telemedicine.

2. Medico-legal issues and malpractice liability: There are no specific standard operating guidelines for telemedicine practice, thus patient privacy and confidentiality are critical barriers in this practice.

3. Absence of accreditation or regulatory policy of telemedicine: Lack of validating and certifying telemedicine services makes physicians worried about practicing telemedicine.

4. Reluctance of many physicians and people to change the traditional way of practicing medicine and adopt a new model of practice.

5. Lack of definite business models to ensure the sustained proper delivery of telemedicine services.

In view of the above, the government and authorities should address these barriers and try to provide solutions in order to achieve a wider implementation of telemedicine, which will have a significant positive impact on the healthcare system in all countries.

## Conclusions

Telemedicine can be an integral part of the healthcare system. Although telemedicine cannot fully substitute the in-person face-to-face consultations or emergency conditions, its proper implementation can ensure efficient, cost-effective, and reliable healthcare delivery to remote underserved areas. It is also a valuable learning tool for physicians all over the world by providing an opportunity for continued medical education and research. Telemedicine can play a crucial role in pandemics, so its application during this era of the COVID-19 pandemic can minimize the burden on the healthcare system and reduce the risk of spreading the virus.

Several obstacles prohibit our countries from enjoying the advantages of telemedicine. The government and relevant authorities must take these issues into consideration. Everyone has the right to obtain a proper level of healthcare, regardless of whether they live in a large city or a distant rural location, and telemedicine can assist us in efficiently achieving this goal.
